# Initial Development of the Early Intensive Behavioral Intervention Parental Self-Efficacy Scale: A Pilot Study

**DOI:** 10.1155/2017/9512180

**Published:** 2017-05-31

**Authors:** Aaron Blocher-Rubin, Paige Krabill

**Affiliations:** ^1^Arizona Autism United, 5025 E. Washington St. #212, Phoenix, AZ 85034, USA; ^2^Capella University, Minneapolis, MN, USA

## Abstract

Early Intensive Behavioral Intervention (EIBI) is an effective treatment for children with autism. However, it is known that some parents struggle to fully implement the program, and providers are not always able to identify the specifics of each family's individualized challenges. The purpose of this pilot study was to begin the process of developing a new instrument, the EIBI Parental Self-Efficacy (EPSE) Scale, to help providers better assess and assist parents in regard to EIBI implementation. The methodology included four phases: scale construction, expert review, pretest administration, and a large sample pilot study (*N* = 192). The final 29-item EPSE Scale contained strong reliability properties (Cronbach's alpha = .900). Factor analysis established five subscales:* Family Well-Being*,* Preparing for Successful Sessions*,* Team Participation*,* Not Giving Up*, and* Working with your Child*. Following this pilot study, future research is recommended to refine and validate the EPSE Scale as a useful clinical tool for EIBI providers.

## 1. Introduction

This pilot study was conducted as a first step toward developing a new instrument, the EIBI Parental Self-Efficacy (EPSE) Scale. The purpose of the EPSE Scale is to assess parents of children with autism who are actively receiving Early Intensive Behavioral Intervention (EIBI), specifically in regard to any challenges they might be experiencing related to the demands of implementing an in-home EIBI program.

Research has repeatedly demonstrated that EIBI, a comprehensive treatment package based on the science of applied behavior analysis (ABA), is currently the most effective evidence-based treatment for young children with autism [1–5]. Although EIBI is known to be highly effective, it is also known that implementation can place heavy demands on parents [6–8]. EIBI is mostly home-based and requires thousands of one-to-one instructional hours with the child for several years, along with ongoing intensive parental involvement expectations. As such, EIBI implementation can also require major lifestyle adjustments for the whole family [8–10].

The ability of parents to be involved is key to the EIBI treatment approach [11, 12]. However, requiring too much from parents can actually be detrimental toward overall intervention effectiveness [13]. Providers need to be keenly aware of each parent's current capacity in order to determine an appropriate balance of expectations. Therefore, the potential value of the EPSE Scale is that it gives providers an objective method to identify any underlying problems experienced by parents, which may not be immediately obvious but could inhibit treatment effectiveness if not properly addressed.

### 1.1. Clinic-Directed versus Community-Based EIBI

As EIBI has evolved into a broad spectrum of intervention packages for children with autism over the past few decades, several publications have helped clarify its core elements [5, 8, 14, 15].* Early* signifies that intervention should begin as soon as possible, typically following diagnosis and preferably by the age of 3.* Intensive *indicates that direct treatment hours should range from about 20 to 40 per week for at least two years, depending on the child's age and other factors.* Behavioral *means that all teaching methods are consistent with the science of ABA and guided by an individualized, developmental, and comprehensive curriculum that addresses a wide range of skills in all domains of functioning.* Intervention *refers to the entire treatment package, which involves creating a structured and predictable learning environment for the child to respond appropriately and be reinforced. Each child's treatment program must be designed and directed by a certified behavior analyst and delivered mostly in a one-to-one context by a team of trained paraprofessional behavioral therapists (i.e., technicians) in the child's natural environment.

Depending on available providers and funding sources, families may receive either a robust clinic-directed model with all services managed by an EIBI provider agency or a community-based model in which parents are responsible for significantly more program management duties [16, 17]. Although there are many variations of community-based models, the general distinctions are that far fewer resources are available and a significant portion of treatment management becomes the parents' responsibility [13, 15]. Staff typically receive less training and supervision, which requires parents to take a more active supervisory role. At times, parents must also recruit their own therapists from the community, as provider agencies may not always have enough capacity to meet demand. Funding is generally much lower than a clinic-directed model and supervisors have less time to spend on each case due to higher caseloads, which can result in diluted treatment with fewer research-based protocols. Parents may need to guide their teams between consultation visits from program supervisors, without necessarily knowing how to respond optimally to various challenges. It is important to note that studies do show that community-based EIBI is effective, but considerably less so than clinic-directed programs [2, 15, 18–20]. The benefit, however, is that community-based models can reach far more children and families in need. Therefore, as the need for EIBI grows, it is critical that providers have better tools to deliver more effective services in community-based models.

### 1.2. Demands on Parents

Implementing EIBI inevitably requires families to manage a wide range of tasks and challenges, especially within a community-based model. For example, parents must establish a robust in-home therapy environment in the context of their daily lives that involves frequent visits from therapists. They typically must be present for up to two sessions per day of about three hours each, in addition to weekly or monthly meetings and training with their therapy team [1, 15, 16, 21, 22]. Some programs require parents to act as cotherapists themselves, in varying capacities from highly structured to naturalistic, which can be stressful for some. Outside of direct therapy time, parents are usually tasked with implementing consistent behavioral strategies with their child, which at times may increase behavioral challenges and require extensive perseverance under pressure [8, 13, 23]. Sadly, these techniques are too often misunderstood by extended family or community members, leaving parents at risk for feeling stressed, socially unsupported, or judged as unable to control their child's behavior despite doing all they can to follow the EIBI program's recommendations [24–26].

Several studies have investigated how well families adapt to EIBI. One study interviewed parents that completed EIBI for at least two years, who believed it was beneficial but also had struggles [7]. The lack of in-home privacy was often difficult and not always respected by therapists. Administrative duties could also be stressful and take time away from the rest of the family. Additional frustration ensued when therapists had issues with reliability, punctuality, and maintaining confidentiality. The authors suggest that EIBI may at times exacerbate marital strain. Another study found that EIBI can be emotionally difficult for mothers, involving fatigue and a sense of sacrifice [6]. Some found it difficult to be both mother and therapist, feeling less effective than other therapists and struggling to transition back into the parent role, in addition to finding time for their other children. A separate study found that EIBI impacted the family's home environment, with requirements such as allocating space exclusively for therapists to work without distractions, and some families had little time for other social activities [10]. Each of these studies identifies different struggles that families have faced, yet the participants only represent parents who were able to complete the EIBI program. The impact of struggles faced by families who were unable to sustain the treatment is still unknown, which further emphasizes the need for a standardized assessment tool to identify risks before they significantly impede treatment implementation.

### 1.3. The Role of Self-Efficacy

Since it is known that EIBI implementation can pose significant challenges for parents, the logic follows that the odds of treatment success could be improved if providers have a way to predict which specific tasks and behaviors are most likely to be a struggle for any given family, so that they can give extra attention to those areas. In the psychological literature, a commonly measured predictor of behavior is self-efficacy, which is defined as the degree to which a person believes he or she is able to perform a particular skill or behavior [27, 28]. A person's level of self-efficacy for any given behavior serves as a predictor because it influences whether or not he or she will engage in that behavior, as well as persist, when confronted by challenges. For that reason, the EPSE Scale is constructed as a measure of self-efficacy for tasks and behaviors specifically related to EIBI implementation, to assess how likely parents are to continue completing those tasks over time as the treatment progresses.

According to Bandura [27], self-efficacy is most relevant when people have the skills, incentive, and opportunity to do the task, so that self-efficacy is the only remaining factor to alter their behavior. Parents implementing EIBI clearly have the incentive and opportunity, but some may not have adequate levels of self-efficacy for all necessary tasks to persist when circumstances are challenging. Utilizing self-efficacy as the guiding psychometric for the EPSE Scale is fitting because self-efficacy has been shown to predict positive parenting practices under stressful circumstances [29]. For the purposes of this study, EIBI implementation is conceptualized as a component of parenting because all parents are responsible for coordinating any treatment necessary for their child's well-being. It is also worthy to note that self-efficacy has been the construct of choice for domain measurement with many other conceptually similar scales, such as assessing one's ability to self-manage a treatment regimen for other health conditions [30, 31].

### 1.4. The Need for a New Instrument

As discussed, EIBI is a very unique and intensive treatment. The specific demands on parents are not easily comparable to other early intervention services that consist of far fewer hours and in general a much less comprehensive approach. No other measurement tool has been published that specifically targets a parent's ability to carry out the tasks and behaviors uniquely related to EIBI implementation, even though it is the most evidence-based treatment available for young children with autism. One other publication, the Early Intervention Parenting Self-Efficacy Scale [32], appears similar on the surface but is not specific to autism or EIBI. Instead, it measures self-efficacy related to feeling competent as a parent and helping the child make progress, while receiving early intervention services such as speech and occupational therapy. This scale may have value for the same target population for which the EPSE Scale is intended, but it measures an entirely different construct and would not be appropriate to specifically identify EIBI treatment implementation challenges.

Bandura [28] emphasizes that self-efficacy measurement tools must be designed to examine specific domains of functioning. It is not useful (or accurate) to simply say that someone has a high level of self-efficacy, for example, because it is not a global construct that applies equally to everything a person does. Instead, the recommended approach to developing self-efficacy based scales is to measure a defined class of performances under various classes of relevant conditions. The EPSE Scale achieves this goal by assessing common tasks specifically associated with implementing EIBI in a variety of circumstances that parents are known to experience. Additionally, because self-efficacy beliefs can vary over time [28], the EPSE Scale is designed to be administered at multiple times throughout the EIBI treatment process as needed so that new concerns can be identified and addressed anytime. The intended outcome is for providers to use the resulting information to facilitate discussion with parents to find solutions and improve their self-efficacy in those areas. In theory, this process should ultimately help families overcome EIBI challenges because improving self-efficacy can change the perception of treatment barriers [33].

For decades, researchers have focused on demonstrating EIBI as an effective treatment, but only recently are researchers noting a need for parental assessment to understand their readiness for such a taxing treatment regimen [12, 34–36]. Currently, EIBI providers do not typically assess barriers experienced by families that affect implementation and adherence [37]. Instead, it is often just assumed that parents can fulfill their requirements [35]. Since not all families complete the recommended treatment time to maximize EIBI outcomes [38], researchers must identify more effective methods to support parents with successful ongoing implementation, especially in community-based models.

Challenges faced by families are an individualized combination of circumstances. Schreibman [39] has noted that practitioners need to be more responsive to feedback from families, as EIBI requires the involvement of the child's total environment. The overall rationale for this study was to develop a tool to identify what each individual family is facing on a case-by-case basis as they attempt to implement EIBI in their home, so that solutions can be equally individualized. The goal of the EPSE Scale is to provide a practical tool to assess parental capabilities related to completing tasks and behaviors necessary for EIBI implementation, which in turn gives providers a clearer insight into what they need to focus on to help each family succeed, thereby adding to the overall social validity of EIBI.

## 2. Methods

This study was completed in four phases: (a) scale construction, (b) expert review field test, (c) pretest administration, and (d) large sample pilot study. The first step involved creating and revising items using best practice guidelines [40]. This draft was shared with subject matter experts for suggestions on individual items and the scale as a whole. The second draft was used for pretest administration with a small sample of the target population, followed by additional feedback to assist with making further improvements. The final phase involved 192 participants completing the scale and providing demographic information. Statistical analyses were conducted to establish key scale properties including reliability, validity, and factor structure. In summary, this pilot study completed a rigorous development and initial testing process for the EPSE Scale, as a first step toward preparing it for future use in research and practice.

### 2.1. Scale Construction

Two primary sources were used to guide the development of the scale's first draft. DeVellis [40] provides a comprehensive overview of best practices for scale development in the social and behavioral sciences, while Bandura [41] offers specific guidelines for developing self-efficacy based scales. As per DeVellis, initial scale construction is done in three steps: (a) determining item format, (b) brainstorming a large pool of possible items, and (c) refining the initial item list.

As recommended by Bandura [41], the items on the EPSE Scale ask participants to rate how certain they are that they can do a given task or behavior under various challenging conditions, consistent with the construct of self-efficacy. The phrasing of items follows a consistent format to avoid confusion. Participants respond using a Likert scale to indicate level of belief in their capabilities, consistent with Bandura's approach and other widely used scales [40]. Each item describes a specific task or behavior associated with implementing EIBI. Although Bandura recommends a scoring range of 0–100, DeVellis cautions against false estimates of precision. Therefore, a 7-point scale is used instead, which DeVellis identifies as a standard approach for measuring attitudes and beliefs. This range also provides enough response variation to conduct robust reliability testing and factor analysis.

A series of best practice guidelines [40] were followed to optimize the initial list of possible items, including testing the reading level to ensure it was not above seventh grade, avoiding any ambiguous words or phrases, and limiting each item to a single task or behavior. Since parental responsibilities associated with EIBI are broad, categories were established based on the literature review to ensure that items were brainstormed within all facets of the domain for a wide range of possible situations.

The literature review followed a comprehensive process involving a wide variety of search terms related to autism, EIBI, treatment efficacy in real world settings, parental adherence and experiences, barriers to treatment, community-based and clinic-directed models, and the role of self-efficacy. Robust online databases were utilized, and references within retrieved articles were analyzed for additional leads as part of a snowball approach. Over 200 peer-reviewed journal articles and several dozen textbook chapters were examined in all. Content was categorized and coded to identify the most common and relevant themes for scale construction.

Using this information, an initial list of 343 items was developed and then refined by identifying items that most clearly represented a range of tasks related to EIBI implementation. The primary considerations were avoiding redundancy, picking items most relevant to the construct, and eliminating wording confusion or vagueness. When finished, the first draft of the scale contained 100 items and a set of instructions that were created in accordance with Bandura's [41] guidelines of clarifying that participants are to rate their perceived capabilities now, rather than what they expect to be able to do in the future.

### 2.2. Expert Review Field Test

The field test followed best practices [40] and included 14 subject matter experts from the following categories: (a) behavior analysts with extensive EIBI experience; (b) parents who had completed EIBI and helped other families; and (c) published researchers in the fields of autism, EIBI, and self-efficacy. Experts were asked to rate each item on the scale as excellent, average, or poor based on the following considerations: (a) readability, (b) relevance, (c) likelihood that people would understand the question, (d) specificity of task or behavior, and (e) representativeness of EIBI parental responsibilities. Comment fields were also available to give feedback on each item, as well as the scale instructions. Experts were also asked for general feedback on (a) how well the tasks and behaviors listed relate to successful EIBI, (b) changes to how the scale should be organized, (c) potential problems, (d) other examples or themes that should be included, (e) concerns for items that might upset or embarrass participants, and (f) other suggestions.

Expert feedback was analyzed to identify common themes and specific suggestions to improve the scale. Ten experts agreed that all of the items represented tasks and behaviors associated with parents successfully implementing EIBI, while four noted that some items did and some did not. Several experts noted that care should be taken to avoid describing any tasks as too difficult, to minimize the risk of parents feeling discouraged.

DeVellis [40] stresses that expert feedback should be strongly considered, but final decisions belong to the researcher as experts may not fully understand the process of scale development. All suggestions were considered in a systematic way by calculating the expert rating scores and categorizing common feedback themes. This input was used to make decisions around revising some items and deleting others. The process concluded with 49 items for the second draft of the EPSE Scale.

### 2.3. Pretest Administration

The pretest administration tested the draft scale with a small sample (*n* = 10) of the target population, to identify any problems before recruiting a large sample and obtain final feedback for revisions. Inclusion criteria were parents or guardians currently implementing EIBI for a child with autism under the age of six. Participants also had to be at least 18 years old, fluent in English, living with the child, and responsible for coordinating services. All recruitment materials directed participants to a website that contained all information, consent forms, and questions for the study. Participation was anonymous. An option was given to provide contact information to participate in a voluntary phone call to provide additional feedback. General questions were asked about the participation experience, including whether the length was reasonable, instructions were clear, the layout was easy, and if they experienced any distress. All items were then displayed again with an optional comment box for participants to share suggestions to improve any particular item.

A simplified item analysis was conducted to observe response patterns, including highest, lowest, and average scores for each item. The range and mean scores were analyzed to determine whether any items were not achieving variation. All items had some variation in scores. After additional revisions based on participant feedback, observations, and reconsideration of scale development guidelines, 29 items were kept as is and five revised, for a total of 34 items.

Participants reported that it took five to 20 minutes to complete the scale, but all felt it was a reasonable amount of time. All stated that the layout was easy to read, they understood what each item was asking, and the rating scale gave them the right amount of response choices. Six participants participated in an optional phone interview and said they felt the scale was easy to complete with straightforward questions. No problems with the scale or experiences of distress or embarrassment were reported. Participants felt they could express their real beliefs and answer all questions accurately. They had no opposition to any of the topics or questions and did not think any items needed to be worded differently to suit the target population.

### 2.4. Large Sample Pilot Study

In the final phase, the EPSE Scale was tested with 192 participants, followed by statistical analyses of psychometric properties to complete final decisions for item elimination. Participants were recruited across the country through many autism organizations and EIBI providers. All materials were available online and participation was anonymous. The website included participant eligibility screening, consent forms, and a demographic survey. For ethical considerations, it was stated clearly that the purpose of the study was to better understand what factors help families succeed with EIBI. It was explicitly noted that the purpose was not to determine whether someone is able to implement EIBI and that it is not yet known whether these items are necessary to be successful. It was also clarified that this scale would not be used in any way to determine eligibility for treatment, in case any participants were concerned that their answers or future use of the scale could have a negative effect on families seeking services.

Following data collection, four major analyses were conducted: (a) initial item analysis, (b) item correlation analysis, (c) reliability analysis, and (d) factor analysis. When all analyses were complete, final decisions were made regarding items to eliminate and factor interpretation, guided by recommendations from several scale development publications [40, 42–44].

## 3. Results

Data analysis involved identifying factor structure, determining items to eliminate, and establishing psychometric properties for the EPSE Scale. The final scale consisted of 29 items and five factors, with exceptionally strong reliability scores. Basic construct and content validity were also established, and an item analysis revealed additional key properties of the scale.

### 3.1. Sample Characteristics

The initial eligibility criteria required that the participant's child be less than six years old and receiving at least 20 hours per week of home-based EIBI, and 41 participants completed the study under these criteria. Unfortunately, the researcher experienced significant recruitment challenges and had to modify the eligibility criteria beyond the intended target population in order to secure enough participants for statistically significant data analysis. A decision was made to expand the parameters to include parents who had limited experience implementing EIBI but could likely relate to the items asked in a meaningful way. Following IRB approval, the age limit was increased to less than nine years old, and hours per week were decreased to five or more. Additionally, parents whose child had received EIBI in the past before the age of nine were accepted, with instructions to respond to the best of their memory regarding how they felt on average while their child was receiving EIBI. The modified sample criteria are clearly the biggest limitation to this pilot study, and all findings should be interpreted accordingly. However, the changes did facilitate more successful recruitment as an additional 151 participants then completed the study for a total of 192, achieving the scale development sample size standards put forth by DeVellis [40].

Participant demographics revealed a broad distribution of household income and employment status, as well as geographical locations from 26 states. The most common categories for respondents were biological parent (97%), over the age of 30 (91%), female (89%), married (78%), and Caucasian (77%). In total, 75% had a child currently receiving EIBI, with 57% meeting the initial eligibility criteria and target population definition of under age six.

### 3.2. Reliability

Reliability was assessed using Cronbach's alpha [45], which is generally recommended for scale development. Cronbach's alpha for the final 29-item EPSE Scale was .900, which is essentially an ideal score according to DeVellis [40]. A split-half analysis was also conducted. Cronbach's alpha for the first half was .852, compared to .865 for the second half. The correlation between forms was .622, and the Spearman-Brown coefficient was .767. Clark and Watson [42] also recommend examining the interitem correlation. The mean interitem correlation for this scale was .245, with a minimum of −.098 and a maximum of .652.

### 3.3. Validity

The process used to develop the EPSE Scale allowed for two forms of basic validity to be established, using standards defined by DeVellis [40]. Construct validity was established by strictly adhering to the guidelines of self-efficacy theory and the boundaries of the scale's primary construct of parental ability to continue implementing EIBI. Content validity was established by conducting the field test, wherein subject matter experts helped determine whether the EPSE Scale was easy to understand, representative of the construct, and appropriate for the target population.

### 3.4. Item Analysis

The item analysis analyzed the mean, standard deviation, variance, range, minimum, and maximum scores. As per DeVellis [40], items with the following characteristics are candidates for elimination: (a) mean near the extremes, (b) low standard deviation, (c) low variance, (d) low range, or (e) minimum or maximum not approaching the extremes.  Table 1 summarizes the mean, lowest, and highest numbers for item scores within each of the identified categories.

Most items had means closer to the maximum than midpoint. Standard deviation and variance averaged above 1.0, and range averaged at least 5. As such, the following criteria were established to mark items for possible deletion: (a) mean above 6.5, (b) standard deviation below 1.0, (c) variance below 1.0, (d) range below 4, (e) minimum above 3, and (f) maximum below 5.

In general, all items had high mean scores. However, since the purpose of the EPSE Scale is to identify struggles, it may not be appropriate to assume that a midpoint score should be the average. One ethical theme from the expert review was that items should not portray EIBI as too difficult, which could discourage families. Therefore, a higher average may be appropriate.

Descriptive statistics were obtained for total scale scores across participants. The range between minimum and maximum scores was 130. The mean scale score was 199.96, with a standard deviation of 20.32, which equals three standard deviations above the midpoint of 136.

The total number of responses for each possible item score was analyzed, and the results revealed a negative skew of −1.288. This result was not surprising since, as previously noted, mean item scores were high, but in general participant data did not display a normal distribution.

For an item correlation analysis, the Pearson product-moment correlation coefficient (*r*) was obtained for all items. Floyd and Widaman [44] state that the minimum score should be 0.20, and Field [43] cautions that items with very high correlations of 0.90 or above may be problematic due to multicollinearity. The matrix revealed no correlations above 0.90, but nearly half were below 0.20. The risk of low correlations is that they may perform poorly in factor analysis. However, as explained in the next section, no such issues arose.

### 3.5. Factor Analysis

Factor analysis identified how many factors were causing item response variation. Items that did not fit into a factor were eliminated. The four major steps conducted were (a) factor extraction, (b) factor rotation, (c) factor loadings, and (d) interpretation of factors.

Factor extraction involves determining how many factors to analyze, using either exploratory or confirmatory methods [40]. Exploratory methods are recommended when there are no firm expectations regarding the number of factors based on theory or prior research [44]. Since the goal of this study was to measure something not previously researched, exploratory factor analysis was utilized. This process was conducted in two ways, using both a principal components analysis (PCA) and a common factor analysis (CFA).

The extraction procedure was conducted with the eigenvalue level set at over one. A scree plot was generated, and the extraction sums of squared loadings for all eigenvalues over 1.0 were examined, separated by PCA versus CFA. The eigenvalue rule [46] states that factors with eigenvalues of less than 1.0 should not be retained. However, DeVellis [40] explains that 1.0 is equivalent to the effect of a single item, so a value just above 1.0 may not be worth retaining either. For this test, eigenvalues showed six factors clearly above 1.0 with the lowest at 1.273, followed by 1.098. Therefore, only the top six factors were retained.

Field [43] also recommends checking the value for the Kaiser-Meyer-Olkin (KMO) measure of sampling adequacy. A value greater than 0.5 signifies that the sample size is adequate for factor analysis, and the KMO value for this sample was .863.

The next step was to conduct factor rotation so that meaningful groupings of items could emerge. Both orthogonal and oblique rotations were conducted with PCA and CFA analyses. Floyd and Widaman [44] state that a high cumulative percentage such as 80% is ideal, so that these factors are accounting for most of the variance. These analyses did not quite achieve that goal, as PCA rotation represented 55.5% of the variance and CFA represented 46.4 percent.

In total, factor extraction and rotation were conducted in four ways: (a) PCA orthogonal, (b) CFA orthogonal, (c) PCA oblique, and (d) CFA oblique. The factor loading results for each combination were analyzed, and CFA orthogonal provided the clearest factor structure.

Several considerations were important to arrive at this conclusion. First, the oblique rotations did not provide enough clarity. Both PCA and CFA factor loading results showed at least seven items that did not meet the minimum strength requirements of .400 for PCA or .350 for CFA. Deleting these items solely on this basis would have put the scale at risk as more items may have been deleted later for other reasons, resulting in potentially too few items overall to accomplish the scale's goals. Additionally, both oblique analyses contained one factor with no items even achieving a minimal loading requirement of .300. Floyd and Widaman [44] state that factor loadings are most meaningful when they exceed .300 or .400 and that at least three items should have significant loadings to retain a factor. Clark and Watson [42] state that factors should load at least .350 in CFA or .400 in PCA and that items loading strongly on only one factor are the best candidates to keep.

By contrast, both orthogonal extractions had strong results with almost all items clustering clearly around all six factors with good distribution. PCA, however, did not clearly reveal which items should be deleted, as too many items loaded strongly on more than one factor. The only area in which PCA outperformed CFA was total variance explained (55.5% versus 46.4%, resp.). However, the initial eigenvalues showed that 14 factors would be required to reach the desired goal of 80 percent, which would be too many for the practical purposes of this scale and would contain six eigenvalues below 1.0.

The process of interpreting factors involves qualitatively examining items loading on each factor. If similarities can be explained as indicators of a theoretical construct, the factor can be interpreted [40]. Loading values were considered as well, with scores below .350 eliminated. Upon examining the items, five factors were interpreted as follows: (a)* Family Well-Being*, (b)* Preparing for Successful Sessions*, (c)* Team Participation*, (d)* Not Giving Up*, and (e)* Working with Your Child*. A sixth factor,* Motivating Others*, was also identified but later eliminated as it only contained two items.

All items for* Family Well-Being *allude to promoting healthy relationships and a balanced life for family members. The* Preparing for Successful Sessions* items focus on daily procedures to facilitate operational aspects of EIBI. The elements of* Team Participation *reflect parent involvement in the design and implementation of the child's program and working with team members.* Not Giving Up* includes items presenting challenges that require perseverance in the face of obstacles. The items in* Working with Your Child* discuss parents doing activities directly with their child that support the behavior change procedures established in the EIBI program.

After rerunning CFA orthogonal analysis with these five factors, cumulative amount of variance was 46.2 percent, just below the score obtained in the original analysis with six factors, thus maintaining the amount of total variance explained despite eliminating one factor. The final rotated factor loadings are provided in Table 2, along with all items and their interpreted factor themes. Reliability scores for all factors are also shown, ranging from .695 to .835, which rates as acceptable to very good [40]. All items loaded above the CFA significance cutoff of .350 on their identified factor group. Some items loaded above .350 for more than one factor, but all except one loaded highest on the correct factor. This item was included in factor five even though its primary loading was on factor one. As shown in Table 2, both scores were above the defined significance level of .400, but factor five was a better conceptual fit with the interpreted themes. Factor one also had the most items while factor five had the fewest. Thus, the move was made to achieve better overall scale composition. This approach is supported by Field [43], as factor analysis is an exploratory tool to use only as a guide for making decisions.

DeVellis [40] states that item elimination decisions should never be made solely on a statistical basis. Original intent of the scale needs to be considered along with statistical evidence. Following researcher review of all data analysis, five items were eliminated that had undesirable qualities from the item analysis and did not fit into one of the established factors.

The final version of the EPSE Scale contains 29 items within five subscales. The instructions state that the scale is intended for parents/guardians of a child diagnosed with autism (or at-risk), who lives at home with this parent/guardian and is currently receiving home-based EIBI. Participants are asked to read each item and rate how certain they are that they can do the stated task regularly on a scale of 1 to 7, with 1 representing that they can never do it and 7 signifying they can always do it. Tasks that they do now should be scored based on their certainty of being able to continue doing it, whereas items they have not done yet should be scored based on their belief that they could do it regularly if they had to start now.

### 3.6. Limitations

The first major limitation was that the methodology did not allow for robust validity. Two basic forms of validity were demonstrated—content and construct validity. The most powerful form, especially for self-efficacy scales, is predictive validity [40, 41], which involves first administering the scale and then observing future behavior to see if there is a clear correlation. For the EPSE Scale, that would require a longitudinal study to see if scale scores accurately predict ongoing parent struggles. Presumably, families with low scores would be at higher risk for discontinuing or reducing the intensity of EIBI treatment. This limitation was known before the study began, as the scope of this pilot study did not allow for a comprehensive long-term evaluation of participants.

The next major limitation, as discussed previously, was not expected and arose due to recruiting challenges. The initial goal was 150 participants, but the planned strategy yielded only 41. Since the target sample size needed to be met for factor analysis, the criteria were modified so that more people would qualify. Of the final sample, more than half did meet the initial eligibility criteria and defined target population for whom the scale is intended. However, a good portion of the participants did not. The participants who only met the revised criteria were likely to have had a good understanding of the items as they did have related experience with implementing EIBI, but all findings should be considered in light of this significant limitation.

The demographics also revealed some limitations. Nearly 90% of respondents were female, and almost all were biological parents. More than 70% were over the age of 35, more than 75% were Caucasian, and nearly 80% were married to the other biological parent. Less than 10% had household income under $30,000, and all had at least a high school diploma or GED. These proportions suggest a potentially more stable subpopulation with fewer barriers than some parents of children with autism. There was low representation from minorities, single parents, younger parents, and low income families who may be at higher risk for implementation difficulties. The sample was built on participants independently responding to announcements from support groups, providers, or autism organizations. It is possible that these parents were more likely to be actively involved in their child's treatment and thus scored higher on their capability confidence levels. Overall, however, nearly every demographic category did have some participant representation.

## 4. Discussion

The results of this pilot study provide an initial framework for researchers to further develop this new instrument that focuses on understanding parental roles within EIBI treatment. As such, the EPSE Scale is the first of its kind, in that it measures self-efficacy specifically related to the tasks and behaviors that facilitate successful implementation of a very unique and comprehensive autism treatment program. Now that initial development of the scale has been completed, future research is warranted to refine and validate the measure.

The area in which this pilot study offers the most promise is in potential implications for practice. The EPSE Scale was designed with a vision of improving outcomes and family experiences through its use in real world settings, most notably in community-based EIBI models. If additional research successfully validates the scale, it can then be made available to EIBI providers around the world as a tool to help objectively identify any challenges parents are experiencing related to implementation. In practice, use of the EPSE Scale could serve to improve overall communication between parents and providers as part of a commitment to finding family-centered solutions.

The purpose of this scale is not to determine whether a family will succeed with EIBI, but rather to identify any critical areas that are presenting a challenge, so that practitioners have the opportunity to provide assistance proactively and increase the odds of success. This study adds to a growing body of research on how to make EIBI consistently successful in real world and community-based settings.

Once a family has begun implementing EIBI, there are as of yet no defined time intervals regarding when to administer the EPSE Scale. It is clearly not appropriate to administer before treatment begins, as parents will not have any implementation experience to relate to when answering the questions. It does appear to be appropriate early on in the treatment process, such as within the first three months of implementation, and again at various points thereafter (e.g., every six to 12 months). Until further research providers clear recommendations, practitioners should use clinical judgment regarding overall frequency, and it may vary based on the client. However, overuse of the instrument through frequent administrations could be detrimental, as participants could become overly familiar with the questions and exhibit response bias. It is also possible that, in some cases, simply administering the tool once and engaging in follow-up conversation to discuss identified challenges might be enough to establish a relationship of open dialogue and proactive support, which could last throughout the remainder of treatment and not require additional formal administrations of the scale. As stated, further research is necessary to determine the optimal schedule for recurring administrations.

Another limitation of this pilot study is that it does not specify exactly how to interpret any given participant's scale and subscale scores. There is, for example, no identified cutoff point that signifies a problem for either an individual item or a group of items making up a themed subscale. Additionally, this pilot study does not provide evidence-based direction as to what the resulting actions should be if a practitioner assesses a family and finds low scores in certain areas that suggest help is needed. The intended concept is that practitioners would use the scale as a way to objectively collect information from families, followed by a supportive conversation to collaboratively discuss identified challenges. Perhaps modifications can then be made to the treatment protocols as needed with the goal of integrating the program as smoothly as possible into the family's life, thereby maximizing a family-centered approach to EIBI. Additional studies will be necessary to determine how to best interpret variability in scores.

As intended, this pilot study provides multiple opportunities for additional research. The first and most important step is to test the current version of the EPSE Scale with participants that strictly align with the intended target population of children less than six years old who are actively receiving at least 20 hours per week of EIBI. To maximize confidence in the findings, such a study should include a very large sample size, and each demographic characteristic should have significant representation to establish strong evidence for the validity of the tool with all members of the target population. As with any scale, overall validation is only achieved over time by repeated use in research with a variety of populations and circumstances [40].

Another critical recommendation is to conduct a longitudinal study that tracks participants for up to several years, to see how scale scores correlate with EIBI treatment outcomes and ongoing challenges. Doing so would assess the highly desired predictive validity of the EPSE Scale, which Bandura [41] cites as the most important property to validate any self-efficacy based scale. If it is found that the scale does have clear predictive power, another question arises as to how far into the future the prediction goes. For example, if the scale identifies a challenge that the family is currently experiencing, does that predict struggles for the next two months, six months, or duration of treatment? How many challenges constitute a risk of early treatment termination? Are there particular combinations of challenges that lead to higher risk? The more that such issues are investigated, the more valuable the EPSE Scale will become in real world settings.

Researchers should also examine what types of practitioner response procedures are most effective. The assumption made here is that practitioners could use scale response data to initiate constructive dialogue with parents about possible solutions. For example, perhaps the EIBI program could be modified to better fit the family's needs, or other community resources could be secured to help struggling families. The general principle is that knowing is better than not knowing, and addressing a problem together will ultimately improve the likelihood of positive treatment outcomes and experiences. That being said, however, it would be ideal to know how to best alleviate a problem once it has been discovered. For that reason, it is recommended that future researchers investigate optimal responses for each EPSE Scale item or domain.

If other studies pursue the recommendations noted thus far of diverse sampling, predictive power, and how to best help after assessment, researchers could then closely investigate the specifics of scale scores. Individual items and subscales all have a wide range of possible scores, and with enough data it could be possible to identify what to expect for specific scoring ranges. The more clearly this relationship is understood, the easier it will be to establish consistent follow-up procedures that are based on an exact score. From the current study alone, it is not yet possible to determine what constitutes a score within a normal range.

### 4.1. Conclusion

This pilot study completed an initial development and evaluation process for a new instrument, the EIBI Parental Self-Efficacy (EPSE) Scale. This 29-item, five-factor tool measures the perceived self-efficacy for parents implementing home-based Early Intensive Behavioral Intervention (EIBI) for their child with autism. The scale demonstrated a series of sound psychometric properties, including a Cronbach's alpha reliability score of .900.

The four phases of this pilot study followed best practice guidelines for scale development in the social and behavioral sciences, thereby contributing to construct validity. These phases involved item generation and scale construction, a field test with expert reviewers, a pretest administration with the target population, and a large sample pilot study with 192 participants. Factor analysis revealed a clear structure of five factors with at least three items each, which were interpreted as follows: (a)* Family Well-Being*, (b)* Preparing for Successful Sessions*, (c)* Team Participation*, (d)* Not Giving Up*, and (e)* Working with Your Child*. The EPSE Scale is now ready for researchers to conduct additional studies toward validation.

This study also contributed to the literature surrounding challenges that parents face when implementing EIBI. Through the processes of a comprehensive literature review, item development, expert review, and participant data analysis, a significant amount of evidence was obtained that suggests these items and factor themes represent real issues that parents face when trying to implement EIBI for their child. It is clear that providers should never take it for granted that parents are adequately equipped to tackle the varying amount of responsibilities they must embrace in order to sustain a successful treatment experience for their child. Providers need to be sensitive to the obstacles parents may face, and work in partnership with them to ensure a successful treatment experience. Hopefully, tools such as the EPSE Scale will facilitate that process and ultimately improve access to effective treatment for all children and families living with autism.

## Figures and Tables

**Table 1:** Means and ranges for scale item scores.

Statistic	*M*	Lowest	Highest
Mean item scores	5.88	3.98	6.68
Standard deviations	1.17	0.63	1.75
Variances	1.44	0.39	3.06
Ranges	5.41	3	6
Minimum scores	1.59	1	4
Maximum scores	7.00	7	7

*Note*. *M*: mean score.

**Table 2:** Factor loadings with orthogonal rotation for the final five factors.

Factors and items	1	2	3	4	5
*Factor 1: Family Well-Being *(*α = .812*)					
Spend enough time with each of your other family members	.721				
Support each other if EIBI is causing any stress for people in your home	.693				
Make time to celebrate small steps of progress with your family	.659				
Find time to take breaks and take care of yourself	.616				
Find a way to have bonding time with your child, which might be separate from therapy time	.533				
Help your extended family understand why EIBI is so important for your child	.479				
Get your family members to join in on some of the home program activities	.417				

*Factor 2: Preparing for Successful Sessions *(*α = .835*)					
Have your home ready when therapists come over to work with your child		.705			
Get your child ready for each session before the therapist arrives		.670			
Set up your home in a way that works for the program and also works for your family		.570			
Set up areas in your home where therapists can work with your child without interruptions		.548			
Keep all program materials organized so therapists can find what they need		.524			
Make sure therapists feel welcome in your home at all times		.471			
Help everyone who lives with you get used to having the therapists in the home		.461			

*Factor 3: Team Participation *(*α = .792*)					
Take good notes during team meetings and discussions with your program supervisor			.728		
Make sure you have a say in choosing your child's goals and designing the program			.657		
Talk to the supervisor if you have concerns about one of the therapists			.537		
Participate in training with your team so you know how to use all the strategies			.520		
Host meetings in your home with your child's therapy team, at least once a month			.506		
Help therapists plan their daily activities with your child			.445		
Observe what the therapists are doing each day			.415		

*Factor 4: Not Giving Up *(*α = .695*)					
Make EIBI one of your top priorities				.709	
Be willing to change parts of your family's lifestyle so that your child can continue EIBI				.691	
Allow therapy sessions to continue when your child is upset				.373	
Continue EIBI even if your child's school is not supportive of it				.356	

*Factor 5: Working with Your Child *(*α = .738*)					
When working with your child, be consistent with what you have been trained to do, even if your child gets upset					.636
Be one of your child's therapists, and balance this role with also being a parent					.578
Be persistent and try different things to get your child to engage in activities with you					.578
Be enthusiastic when you work with your child, even if you feel sad about other things	.550				.473

*Note*. Varimax rotation procedure used in SPSS. Numbers across header row represent extracted factors. Item loadings lower than the assigned factor are not shown. Cronbach's alpha scores for each factor are provided.

## References

[1] Eldevik S., Hastings R. P., Hughes J. C., Jahr E., Eikeseth S., Cross S. (2009). Meta-analysis of early intensive behavioral intervention for children with autism. *Journal of Clinical Child & Adolescent Psychology*.

[2] Flanagan H. E., Perry A., Freeman N. L. (2012). Effectiveness of large-scale community-based intensive behavioral intervention: a waitlist comparison study exploring outcomes and predictors. *Research in Autism Spectrum Disorders*.

[3] Peters-Scheffer N., Didden R., Korzilius H., Sturmey P. (2011). A meta-analytic study on the effectiveness of comprehensive ABA-based early intervention programs for children with Autism Spectrum Disorders. *Research in Autism Spectrum Disorders*.

[4] Reichow B. (2012). Overview of meta-analyses on early intensive behavioral intervention for young children with autism spectrum disorders. *Journal of Autism and Developmental Disorders*.

[5] Virués-Ortega J. (2010). Applied behavior analytic intervention for autism in early childhood: meta-analysis, meta-regression and dose-response meta-analysis of multiple outcomes. *Clinical Psychology Review*.

[6] Granger S., Des Rivières-Pigeon C., Sabourin G., Forget J. (2012). Mothers’ reports of their involvement in early intensive behavioral intervention. *Topics in Early Childhood Special Education*.

[7] Grindle C., Kovshoff H., Hastings R., Remington B. (2009). Parents' experiences of home-based applied behavior analysis programs for young children with autism. *Journal of Autism & Developmental Disorders*.

[8] Love J. R., Carr J. E., Almason S. M., Petursdottir A. I. (2009). Early and intensive behavioral intervention for autism: a survey of clinical practices. *Research in Autism Spectrum Disorders*.

[9] Cebula K. R. (2012). Applied behavior analysis programs for autism: Sibling psychosocial adjustment during and following intervention use. *Journal of Autism and Developmental Disorders*.

[10] Trudgeon C., Carr D. (2007). The impacts of home-based early behavioural intervention programmes on families of children with autism. *Journal of Applied Research in Intellectual Disabilities*.

[11] Sheinkopf S. J., Siegel B. (1998). Home-based behavioral treatment of young children with autism. *Journal of Autism & Developmental Disorders*.

[12] Solish A., Perry A. (2008). Parents' involvement in their children's behavioral intervention programs: parent and therapist perspectives. *Research in Autism Spectrum Disorders*.

[13] Smith T., Buch G. A., Gamby T. E. (2000). Parent-directed, intensive early intervention for children with pervasive developmental disorder. *Research in Developmental Disabilities*.

[14] Behavior Analyst Certification Board (2012). *Guidelines, Health Plan Coverage of Applied Behavior Analysis Treatment for Autism Spectrum Disorder*.

[15] Perry A., Cummings A., Dunn Geier J. (2008). Effectiveness of intensive behavioral intervention in a large, community-based program. *Research in Autism Spectrum Disorders*.

[16] Butter E. M., Wynn J., Mulick J. A. (2003). Early intervention critical to autism treatment. *Pediatric Annals*.

[17] Reichow B., Wolery M. J. (2009). Comprehensive synthesis of early intensive behavioral interventions for young children with autism based on the UCLA young autism project model. *Journal of Autism and Developmental Disorders*.

[18] Boyd R. D., Corley M. J. (2001). Outcome survey of early intensive behavioral intervention for young children with autism in a community setting. *Autism*.

[19] Cohen H., Amerine-Dickens M., Smith T. (2006). Early intensive behavioral treatment: Replication of the UCLA model in a community setting. *Journal of Developmental and Behavioral Pediatrics*.

[20] Magiati I., Charman T., Howlin P. (2007). A two-year prospective follow-up study of community-based early intensive behavioural intervention and specialist nursery provision for children with autism spectrum disorders. *Journal of Child Psychology Psychiatry*.

[21] Green G., Brennan L. C., Fein D. (2002). Intensive behavioral treatment for a toddler at high risk for autism. *Behavior Modification*.

[22] Tzanakaki P., Grindle C., Hastings R. P., Hughes J. C., Kovshoff H., Remington B. (2012). How and why do parents choose early intensive behavioral intervention for their young child with autism?. *Education and Training in Autism and Developmental Disabilities*.

[23] Howard J. S., Sparkman C. R., Cohen H. G., Green G., Stanislaw H. (2005). A comparison of intensive behavior analytic and eclectic treatments for young children with autism. *Research in Developmental Disabilities*.

[24] Jensen V. K., Sinclair L. V. (2002). Treatment of autism in young children: behavioral intervention and applied behavior analysis. *Infants Young Children: An Interdisciplinary Journal of Special Care Practices*.

[25] Moore T. R., Symons F. J. (2011). Adherence to treatment in a behavioral intervention curriculum for parents of children with autism spectrum disorder. *Behavior Modification*.

[26] Phetrasuwan S., Shandor Miles M. (2009). Parenting stress in mothers of children with autism spectrum disorders. *Journal for Specialists in Pediatric Nursing*.

[27] Bandura A. (1977). Self-efficacy: toward a unifying theory of behavioral change. *Psychological Review*.

[28] Bandura A., Brennan S. F., Hastings C. (1997). The nature and structure of self-efficacy. *Self-Efficacy: The Exercise of Control*.

[29] Coleman P. K., Karraker K. H. (1998). Self-efficacy and parenting quality: findings and future applications. *Developmental Review*.

[30] Bijl J. V. D., Poelgeest‐Eeltink A. V., Shortridge‐Baggett L. (1999). The psychometric properties of the diabetes management self-efficacy scale for patients with type 2 diabetes mellitus. *Journal of Advanced Nursing*.

[31] Horan M. L., Kim K. K., Gendler P., Froman R. D., Patel M. D. (1998). Development and evaluation of the osteoporosis self-efficacy scale. *Research in Nursing and Health*.

[32] Guimond A. B., Wilcox M. J., Lamorey S. G. (2008). The early intervention parenting self-efficacy scale (EIPSES): scale construction and initial psychometric evidence. *Journal of Early Intervention*.

[33] Hoagwood K. E. (2005). Family-based services in children's mental health: a research review and synthesis. *Journal of Child Psychology and Psychiatry*.

[34] Osborne L. A., McHugh L., Saunders J., Reed P. (2008). Parenting stress reduces the effectiveness of early teaching interventions for autistic spectrum disorders. *Journal of Autism and Developmental Disorders*.

[35] Remington B. (2010). Early intensive behavioral intervention and family psychological adjustment. *Inside Behavior Analysis*.

[36] Silva L. M. T., Schalock M. (2012). Autism Parenting Stress Index: Initial psychometric evidence. *Journal of Autism & Developmental Disorders*.

[37] Springer C., Reddy L. (2004). Measuring adherence in behavior therapy: opportunities for practice and research. *The Behavior Therapist*.

[38] Lord C., Wagner A., Rogers S. (2005). Challenges in evaluating psychosocial interventions for autistic spectrum disorders. *Journal of Autism and Developmental Disorders*.

[39] Schreibman L. (2000). Intensive behavioral/psychoeducational treatments for autism: research needs and future directions. *Journal of Autism and Developmental Disorders*.

[40] DeVellis R. F. (2012). *Scale Development—Theory and Applications*.

[41] Bandura A., Urdan T., Pajares F. (2006). Guide to constructing self-efficacy scales. *Self-Efficacy Beliefs of Adolescents*.

[42] Clark L. A., Watson D. (1995). Constructing validity: basic issues in objective scale development. *Psychological Assessment*.

[43] Field A. (2005). *Discovering Statistics Using SPSS*.

[44] Floyd F. J., Widaman K. F. (1995). Factor analysis in the development and refinement of clinical assessment instruments. *Psychological Assessment*.

[45] Cronbach L. J. (1951). Coefficient alpha and the internal structure of tests. *Psychometrika*.

[46] Kaiser H. F. (1960). The application of electronic computers to factor analysis. *Educational and Psychological Measurement*.

